# Supplementary material for: Sex-structured disease transmission model and control mechanisms for visceral leishmaniasis (VL)
Source: PLoS One. 2024 Apr 2;19(4):e0301217. doi: 10.1371/journal.pone.0301217 (PMC10986940; doi:10.1371/journal.pone.0301217)
Supplement: Attachment — Submitted filename: Response to reviewers.pdf [file pone.0301217.s001.pdf]

Response to reviewers' comments and questions

Title of the paper: **Sex-structured disease transmission model and control mechanisms for Visceral leishmaniasis (VL).**

Journal: **Plos One**

Authors: **Temesgen Debas Awoke, Semu Mitiku Kassa, Kgomotso Suzan Morupisi, and Gizaw Mengistu Tsidu.**

First of all, we thankfully appreciate the anonymous reviewers for their valuable suggestions that gave us a chance to improve our previously submitted paper.

The following paragraphs include both the reviewers' comments and our corresponding response. Details are as follows:

Reviewer # 1:

1. I am wondering about the fitted values. With the small number of data and fit many parameters, I think it is hard to get the appropriate results. Please clarify with the confidence interval.

Response from Authors: We are thankful to the reviewer for pointing out this important issue. We accepted the comment and revised the whole contents of the manuscript based on this comment. We have exhaustively searched related parameter values from the literature to reduce the number of model parameters to be fitted (see the improved values in Table 4 on page 18). However, as it was mentioned in the manuscript, there is a very limited age-structured time series data related the VL dynamics. This forced us to fit most of our model parameters from the existing data.
However, to improve the results and get better estimates with small data, we employed the bootstrapping technique and obtained the confidence intervals for each of the estimated parameters. This is now incorporated in the revised manuscript (please see Table 4 on page 18).

2. The numerical solutions are unclear. Please provide an explanation for each figure not only stating the results but also the implication of the results.

Response from Authors: Thanks again for the comment. We still feel that the numerical solutions are explained corresponding to each figure and their implications are also indicated. However, to address the concern of the reviewer, we have now added explanations for some of the figures. The improved statements are highlighted in blue color in the revised manuscript.

Additional Editor Comments:

The reviewer raises concerns about parameter fitting and I agree. While the authors performed a sensitivity analysis, the model is prone to overfitting due to so many parameters and so little data points. As the reviewer suggest, please provide confidence intervals. Also, for as many parameters as possible, look for values in the medical/biological literature. For example, is the loss of immunity (psi_r and psi_h) or the incubation period (eta and phi) truly so different for males and females? Or is it just a consequence of parameter fitting? To me, such a difference sounds unrealistic. At the same time I am confident that even if you set those parameters to any reasonable number you find in the literature and then fit the remaining parameters to your data, you will still see a good fit.

Response from Authors: We would like to thank the editor for the suggestions, comments, and questions that gave us a chance to improve our previously submitted paper. Applying different rates for loss of immunity and the incubation period is not the consequence of parameter fitting. The assumptions were made based on evidence and discussion from literature (see References 24, 30, 42). However, accepting the comment and considering its importance in slightly reducing the number of model parameters to be estimated, we have now revised the whole content of the manuscript based on this suggestion. Research conducted so far in relation to VL are not classified based on sex. Hence, we could not obtain data for most of our model parameters from the literature. We minimized the number of fitted model parameters based on the information we got from the literature (please see Table 4 page 18). All revised symbols and statements are highlighted in blue color. As suggested, we now calculated and included the confidence interval for each of the fitted parameters.
